# Obesity prevalence from a European perspective: a systematic review

**DOI:** 10.1186/1471-2458-8-200

**Published:** 2008-06-05

**Authors:** Anne Berghöfer, Tobias Pischon, Thomas Reinhold, Caroline M Apovian, Arya M Sharma, Stefan N Willich

**Affiliations:** 1 Institute for Social Medicine, Epidemiology and Health Economics, Charité University Medical Center, Berlin, Germany; 2 Department of Epidemiology, German Institute of Human Nutrition (DIfE) Potsdam-Rehbruecke, Germany; 3 Center for Nutrition and Weight Management, Boston University School of Medicine and Boston Medical Center, Boston, MA, USA; 4 Canada Research Chair in Cardiovascular Obesity Research and Management, McMaster University Medical Center, Hamilton, Ontario, Canada

## Abstract

**Background:**

Obesity has been recognised as an important contributing factor in the development of various diseases, but comparative data on this condition are limited. We therefore aimed to identify and discuss current epidemiological data on the prevalence of obesity in European countries.

**Methods:**

We identified relevant published studies by means of a MEDLINE search (1990–2008) supplemented by information obtained from regulatory agencies. We only included surveys that used direct measures of weight and height and were representative of each country's overall population.

**Results:**

In Europe, the prevalence of obesity (body mass index ≥ 30 kg/m^2^) in men ranged from 4.0% to 28.3% and in women from 6.2% to 36.5%. We observed considerable geographic variation, with prevalence rates in Central, Eastern, and Southern Europe being higher than those in Western and Northern Europe.

**Conclusion:**

In Europe, obesity has reached epidemic proportions. The data presented in our review emphasise the need for effective therapeutic and preventive strategies.

## Background

"Let me have men around me that are fat..."

In the first act of Shakespeare's *Julius Caesar*, the Roman emperor suggests that higher body weight correlates with a well-balanced mental disposition. In Caesar's times, of course, obesity was not considered a medical risk factor. Since the nineteenth century, however, a high-calorie diet together with a sedentary lifestyle has been recognised as a potential risk factor for cardiovascular disease [1], cancer, and diabetes mellitus [2]. A variety of factors influence the rate of obesity in any particular region, including age patterns [3], socioeconomic factors [4], and a lack of physical activity [5]. In the medical community and at public health institutions worldwide, awareness is growing of the need to develop and implement effective treatments for obesity [6-8].

To date there have been several studies on the prevalence of obesity in Europe, most of which have involved national or regional cross-sectional surveys. There have also been international surveys, such as the WHO Multinational Monitoring of Trends and Determinants in Cardiovascular Diseases (MONICA) and the WHO Countrywide Integrated Noncommunicable Diseases Intervention (CINDI). However, some of these studies are based only on self-reports and may thus provide biased estimates of the prevalence of obesity in the general population. To help provide a clearer picture of the current situation, we aimed in the present study to summarise the available epidemiological data on the prevalence of obesity in European countries. We investigate the health-economic burden of obesity in a separate paper [9].

## Methods

### Searching

In population-based studies, overweight and obesity are frequently identified and evaluated using the body mass index (BMI), calculated as weight in kilograms divided by the square of height in metres. We used the four classes of increasing severity which the World Health Organisation has defined consistent with the notion of graded risk (BMI 18.5 – 24.9 kg/m^2 ^= normal; 25.0 – 29.9 kg/m^2 ^= pre-obese/overweight; ≥ 30.0 kg/m^2 ^= obese).

For the present study, we performed a systematic MEDLINE search in February 2008 using PubMed. The search limits included the date of publication (i.e. between 1 January 1990 and the start of our MEDLINE search), the age of the subjects (18 years and older), and was limited to human studies only. The search words had to be part of the title or abstract.

For each country on the European continent, we performed a separate search using the following three keyword combinations:

1.) obes* AND country

2.) BMI AND country NOT obes* AND country

3.) adiposity AND country NOT obes* AND country NOT BMI and country

The Boolean operator "NOT" was used to exclude articles that had been found in previous searches. If the number of results for a keyword combination exceeded 200, we refined the search by adding the phrase "AND epidemiol*". We also performed a search based on the references cited in review articles. Other terms related to obesity and BMI, such as weight, excess weight, fatness, body size, etc., were not included as keywords. The risk of overlooking relevant epidemiological data was minimised, however, by scanning for cross-references and by checking review articles. In addition to our systematic PubMed search, we obtained data from the official website of the European Union .

### Selection

To be included in our review the articles had to fulfil the following criteria:

- The age range had to be representative of the respective country's or region's adult population and at least include subjects between the ages of 25 and 65. Representativeness was assumed if the Methods section of the publication indicated that the study population had been randomly selected.

- The studies had to use direct measurements of anthropometric data.

### Validity assessment

We analysed our search results in three rounds. In the first round, we included or excluded articles based on their title. In the second round, we selected or dropped these articles after reviewing their abstracts. In the third round, we obtained the full-text version of each of the remaining articles. Two researchers assessed the publications independently. Disagreements regarding study selection or the methodological assessment of studies were resolved in discussions.

### Study characteristics

We included national surveys and regional surveys. Studies that used interviews or questionnaires to obtain anthropometric data were only included if studies based on direct measures were unavailable. Studies in which the age distribution within the study population was only partially representative of the age distribution in the general adult population of the country or region in question were only included if completely representative data were not available. If several figures were reported from within the same population sample, as is possible in serial surveys, the most recent data were included. If we were unable to find any articles using the abovementioned criteria, we used data obtained from the International Association for the Study of Obesity (IASO) website [10].

## Results

Using the keyword combinations and limits described above, our PubMed search returned a total of 2890 results. Ultimately, 49 articles fulfilled our inclusion criteria. Most of these were cross-sectional surveys, but some were longitudinal cohort studies that estimated the prevalence of obesity using baseline data.

The prevalence of obesity in men ranged from 4.0% to 28.3% and in women from 6.2% to 36.5% (Table 1). The highest prevalences (i.e. greater than 25%) were found in regions of Italy and Spain in both sexes [11,12], as well as in Portugal, Poland, the Czech Republic, Romania, and Albania in women [13-17]. Eastern Europe and the Mediterranean countries showed higher prevalences of obesity than countries in Western and Northern Europe. The time span during which the surveys were conducted varied considerably, the earliest study having been performed in the late 1980s [18] and the most recent in 2005 [19].

**Table 1:** Studies on the prevalence of obesity in Europe over the past fifteen years sorted by country

						**Prevalence of Obesity**	
**Reference**	**Study design**	**Data collection**	**Year of survey**	**Population**	**Sample size and age range**	**Men**	**Women**

[17] #	CSS	dm/q	2001	Albania reg.	n = 1,120 age 25 or older	22.0	30.9
[43] #	CSS	dm/q	1999	Austria reg.	n = 841 age 25 – 64	9.7	13.7
[44] # MONICA	CSS	dm/q	1990–1992	Belgium reg.	n = 912 age 25 – 64	14.7	17.5
[10] #	-	-	1994	Bulgaria	n = 1,996 age 25–74	15.3	20.9
[18] # NHANES	CSS	dm/q	1978–1987	Croatia reg.	n = 4,507 age 18 – 74	10.0	13.6
[45]	CSS	dm	-	Czech Republic reg.	n = 933 age 19 – 60	17.0	18.9
[15] MONICA	CSS	dm/q	1992	Czech Republic nat.	n = 2,353 age 25 – 64	18.6	28.2
[19] HAPIEE #	CSS	dm/q	2002–2005	Czech Republic nat.	n = 7,081 age 45 – 69	30.0	32.0
[10] #	-	-	1999–2000	Cyprus	n = 1,019 age 25 – 64	26.6	23.7
[46] MONICA	CSS	dm	1992	Denmark reg.	n = 1,624 age 30 – 60	13.0	11.0
[47]	PCS	dm/q	1993	Denmark reg.	n = 47,589 age 50 – 64	14.0	13.0
[48] #	CSS+ PCS	dm/q	1991–1994	Denmark reg.	n = 30,482 age 20 – 84	12.3	11.2
[32] #	CSS	dm/q	1997	Estonia nat.	n = 1,154 age 19 – 64	9.9	17.4
[49]	CSS	q	1998	Estonia nat.	n = 2,000 age 20 – 64	10.0	15.0
[50]	CSS	dm/q	1997	Finland nat.	n = 37,940 age 25 – 64	14.0	19.9
[49]	CSS	q	1998	Finland nat.	n = 5,000 age 20 – 64	11.0	10.0
[51] #	PCS	dm/q	1992	Finland nat.	n = 17,441 age 25 – 64	14.2	14.9
[52] #	CSS	q	2003	France nat.	n = 25,770 age 15 or older	11.3*	11.3*
[53]	CSS	q	1994–1996	France reg.	n = 1,169 age 30 – 77	4.0	6.2
[54]	CSS	q	1991	France nat.	n = 15,106 age 20 – 99	6.5	7.8
[55] German National Health Survey 1998 #	CSS	dm/q	1998	Germany nat.	n = 7124 age 18 – 79	18.8	21.7
[56] MONICA	CSS	dm/q	1994–1995	Germany reg.	n = 4,792, 25 – 74	19.3	21.5
[57], [58], [59], [60] ATTICA #	CSS	dm/int	2001–2002	Greece reg.	n = 3,042 age 20 – 89	20.0	15.0
[10] #	-	-	1992–1994	Hungary	n = 2,559 age 18 or older	21.0	21.2
[16] #	CSS	dm/q	1985	Iceland nat.	n = 1,361 age 35 – 64	11.0	11.0
[61], [62] #	CSS	dm/q	1997–1999	Ireland nat.	n = 1,379 age 18 – 64	20.0	16.0
[63]	CSS	dm/q	1990–1991	Ireland reg.	n = 784 age 35 – 64	14.2	22.6
[3] InCHIANTI	CSS	dm	1998–2000	Italy reg.	n = 1,453 age 20 or older	22.0	27.7
[11] CINDI	CSS	dm	1998–1999	Italy reg.	n = 1,200 age 25 – 74	25.2	32.3
[64]	CSS	dm	1998	Italy reg.	n = 856 age 35 – 64	14.6*	14.6*
[32] #	CSS	dm/q	1997	Latvia nat.	N = 2,292 age 19 – 64	9.5	18.3
[32] #	CSS	dm/q	1997	Lithuania nat.	N = 2,096 age 19 – 64	11.4	6.0
[49]	CSS	q	1998	Lithuania nat.	n = 3,000 age 20 – 64	10.0	18.0
[10] #	-	-	-	Luxembourg	n = 5,206 age 16 or older	15.3	13.9
[65], [66] MORGEN #	CSS	dm/q	1993–1997	Netherlands reg.	n = 17,824 age 20 – 59	8.6	9.5
[67] TROMSO #	CSS	dm/q	2001	Norway reg.	n = 7,954 age 29 – 75	18.2	20.8
[14] MONICA	CSS/PCS	dm/q	1992–1993	Poland reg.	n = 1,040 age 35 – 64	22.3	36.5
[19] HAPIEE #	CSS	dm/q	2002–2005	Poland nat.	N = 9,170 age 45 – 69	27.0	34.0
[13] #	CSS	dm/q	-	Portugal reg.	n = 1,436 age 18 – 90	13.9	26.1
[16] #	CSS	dm/q	1985	Romania reg.	n = 1,156 age 35 – 64	20.0	31.0
[68] #	CSS	q	2001	Slovenia nat.	n = 9,043 age 25 – 64	16.5	13.8
[12] EPIC #	PCS	dm/q	1992–1996	Spain nat.	n = 37,663 age 29 – 69	28.3	30.0
[69]	CSS	dm/interview	1998	Spain reg.	n = 1,226 age 18 – 65	25.5	30.7
[70], [71], SEEDO	CSS	dm/q	1990–2000	Spain reg.	n = 9,885 age 25 – 60	13.4	15.8
[72]	CSS	dm/q	1999–2000	Spain reg.	n = 3,179 age 25 – 74	25.6	26.3
[73]	CSS	dm/q	1998–2000	Spain reg.	n = 3,421 age 25 – 60	16.9	20.9
[74]	CSS	dm	1989	Spain reg.	n = 704 age 15 and older	12.0	12.0
[75] INTERGENE	CSS	dm/q	2002	Sweden reg.	n = numbers not given, age 25 – 64	15.0	11.0
[76] MONICA #	CSS	dm/q	1999	Sweden reg.	n = 6,914 age 25 – 74	14.6	15.7
[77] MONICA	CSS	dm/q	1992–1993	Switzerland reg.	n = 3,299 age 25 – 74	15.5	9.8
[78] #	CSS	dm/q	2003	Switzerland reg.	n = 12,271 age 35 – 74	15.0	11.0
[79] #	CSS	dm/q	2003	UK nat.	n = 14,836 age 16 or older	22.2	23.0

CSS = cross sectional survey; PCS = prospective cohort study; dm = direct measurements; q = questionnaire; int = interview, reg. = regional representative data, nat. = national representative data, * no gender strata given, # data used for figure.)

**Figure 1:**
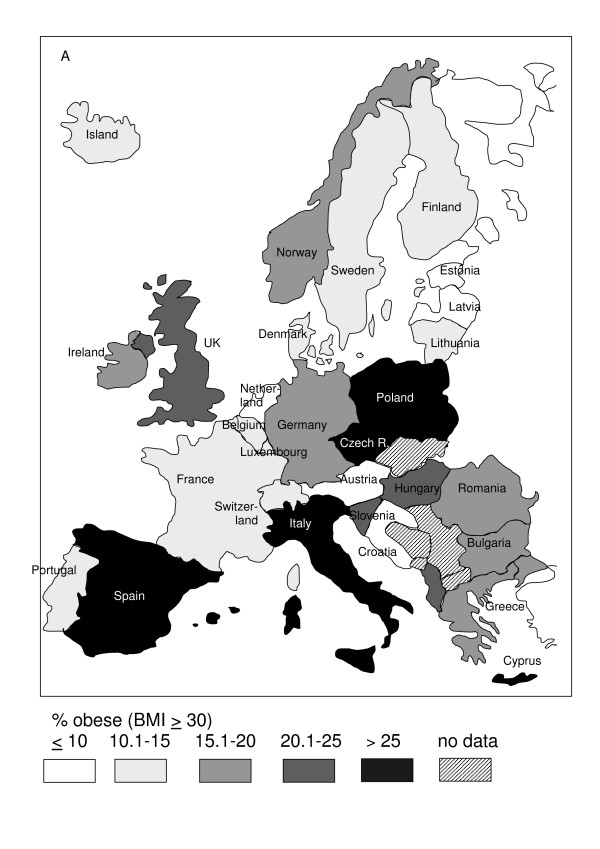
**Regional variation in prevalence of obesity (BMI ≥ 30 kg/m^2^) in men in Europe.** Data in % from Table 1. If more than one study contribute data of different strata, the latest survey was chosen for the map.

**Figure 2:**
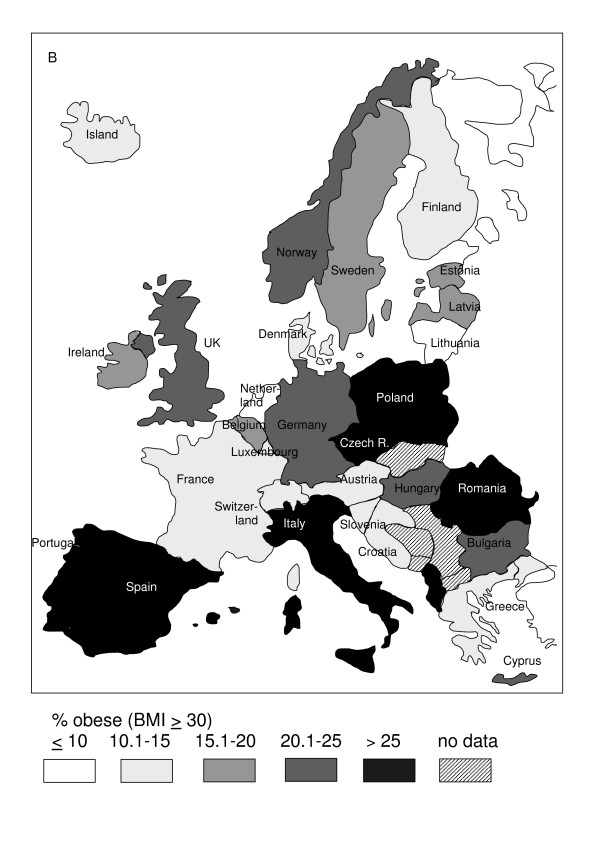
**Regional variation in prevalence of obesity (BMI ≥ 30 kg/m^2^) in women in Europe.** Data in % from Table 1. If more than one study contribute data of different strata, the latest survey was chosen for the map.

Geographic variation in the prevalence of obesity among European countries is shown in Figure 1 for the male population and in Figure 2 for the female population.

## Discussion

Data from the WHO-MONICA study revealed markedly different prevalence patterns within Europe, ranging from 7% in Swedish men to 45% in women from Lithuania [20,21]. In the United States, prevalences comparable to those seen in Europe today were already observed in data from the NHANES III survey, conducted 15 years ago. In a recent NHANES survey, which includes data from 2004, prevalences in the US ranged from 29% in white men to 50% in black women [22]. Data from the US show that the prevalence of obesity is rising continuously, and similar trends have been reported recently for the Chinese population, in which the prevalence of obesity has doubled over the past decade [23]. With these worldwide trends in mind, and based on the data currently available for Europe, it would appear safe to assume that obesity in Europe is approaching, if it has not already reached, epidemic proportions. Naturally, it needs to be noted here that there are populations, such as the Japanese, that do not follow the worldwide trend [24].

For several countries in Europe, there are no data available that go beyond the second MONICA survey conducted in the early 1990s. Other European studies have not contributed nationally representative data based on direct measures: The EPIC cohort (European Prospective Investigation into Cancer and Nutrition) for example, was conducted primarily to investigate an association between cancer and diet and only included subjects between the ages of 50 and 64 [25]. The EURALIM Project [26] aimed to determine the extent to which European data could be pooled in a common database for international comparisons and thus did not collect any new data. The Institute of European Food Studies (IEFS) reported very low prevalences, but used only self-reported obesity as its measure [27]. A series of recent studies in Sweden were based primarily on self-reported weight and height [28,29]. As a result, we were only able to include two studies with regional samples from this series in our survey. Finally, it should be noted that the IASO's International Obesity Taskforce (IOTF) database does not routinely list references and only indicates whether data are based on self-reported weight and height. Moreover, because the IOTF database does not provide information on sample sizes or sampling methods, it is often unclear whether these data are representative.

Most of the studies included in our review were restricted to specific regions or age ranges. Thus for Albania, Austria, Croatia, Denmark, Greece, Italy, Netherlands, Norway, Portugal, Romania, Sweden, and Switzerland, only regional data within countries were available, in part because we had to exclude several national surveys that had been based solely on self-reported weight and height. As a result, our review may overestimate or underestimate the prevalence of obesity in these countries. France is the only country in our review for which data were only available based on self-reports. We included these data in our review because data with greater validity were not available. With regard to Spain, the regional surveys included in our study all had a sufficiently representative sample and adequate sample size. Nevertheless, the data showed large variations, which may be attributable to regional differences in living conditions and socioeconomic factors related to obesity prevalence.

Overall, in the central, eastern, and southern regions of Europe, prevalence rates are higher than in the western or northern regions. This geographic pattern can be explained, at least in part, by different socioeconomic conditions, as well as by lifestyle and nutritional factors. The prevalence of obesity in Spain and Italy, in particular, is high, and there has been recent discussion in the literature about urbanisation and the globalisation of certain lifestyle factors that have had a negative impact on the traditional Mediterranean diet [30].

Aside from dietary patterns, alterations in lifestyle have been identified as major factors contributing to the growing BMI. Data from a large European prospective cohort indicate that the intake of fatty acid fractions accounted for less that 1% of variance in BMI in Spanish subjects, whereas all dietary and non-dietary variables accounted for 21% of variance in BMI among women and 6.7% of variance in BMI among men [12]. Furthermore, an analysis of sedentary and non-sedentary leisure activities in 15 EU countries showed that obesity and overweight are strongly associated with sedentary lifestyle and a lack of physical activity [31]. These results are supported by data from the Baltic States. Approximately 50% of the participants in three national surveys conducted in Estonia, Latvia, and Lithuania in 1997 indicated that they did not engage in physical activity during their leisure-time [32].

The wide variations in BMI seen in different European populations may also be due in part to ethnic affiliation. In studies including various groups of immigrants in Canada [33] and Sweden [34], ethnicity has been shown to be a major determinant of obesity independently of socioeconomic factors. Many European countries have undergone substantial population changes due to immigration from Eastern Europe, as well as from outside of Europe, over the past two decades [35].

The prevalence of obesity in Europe has significantly increased over the past several decades, a phenomenon that is corroborated by data from several other industrialised countries outside Europe. In the mid-1980s, 15% of the male and 17% of the female population in Europe had a BMI ≥ 30 kg/m^2 ^[36], meaning that the rate of obesity has increased by approximately 30% over the past 10 to 15 years. National surveys for example in the United Kingdom indicate that the rate of obesity there increased by approximately 15% between 1943 and 1965 [37].

The surveys included in our review were conducted within a wide time span between the mid-1980s and 2003. As a result, the prevalence data presented here were collected during different phases of an increasing trend. We are aware that the decision only to include surveys based on direct measures decreases comparability over time, because recent surveys based on questionnaires could not included in our review. Nevertheless, we felt that this limitation is outweighed by the increased precision of measurement provided by our approach.

Indeed, the underreporting of weight and height in many surveys yields far lower prevalence rates. This can be seen, for example, in the IEFS survey, which included approximately 15,000 subjects from 15 different countries in the European Union [27,38]. Self-reports of BMI are probably optimistic compared to direct measurements made by an interviewer. Table 2 compares self-reported and directly measured data for those countries for which comparable surveys were published. Although there were considerable discrepancies in several countries, other countries showed prevalences that did not differ markedly between self-report and direct measurement. The prevalences based on self-reported data given for France are far lower than those seen in countries for which prevalence data are based on direct measures. However, because of the discrepancies described above, it would be incorrect to assume that the low prevalences seen in France are due entirely to underreporting. Clearly, life-style factors and other variables may play an important role.

**Table 2:** Studies on the European prevalence of obesity (BMI ≥ 30 kg/m^2^) among the adult population based on self-reported and direct measurements.

		**Self report**			**Direct measure**	
**Population**	**Year of Survey/Reference**	**Men**	**Women**	**Year of Survey/Reference**	**Men**	**Women**

Denmark	2001 [80]	11.8	12.5	1991–1994 [48]	12.3	11.2
Finland	1994 [81]	12.2	12.9	1992 [51]	14.2	14.9
Germany	2002 [82]	12.1	10.3	1998 [55]	18.8	21.7
Greece	2002 [2]	20.1	16.6	2001–2002 [57], [58], [59], [60]	20.0	15.0
Italy*	1991 [83]	7.0	6.1	1998–2000 [3]	22.0	27.7
Lithuania	2002 [84]	16.2	16.0	1997 [32]	11.4	6.0
Netherlands	2001 [85]	8.9	12.4	1993–1997 [65], [66]	8.6	9.5
Portugal	1998–1999 [86]	11.5	14.2	[13]	13.9	26.1
Spain	1993 [87]	8.1	8.2	1992–1996 [12]	28.3	30.0
Sweden	2000–2001 [29]	9.6	9.3	1999 [76]	14.6	15.7
Switzerland	2000 [88]	6.0	4.0	2003 [78]	15.0	11.0
United Kingdom	2000–2001 [89]	22.0	22.0	2003 [79]	22.2	23.0

Data from selected countries, where studies best matching with regard to sample characteristics and survey date were available.

*Sample characteristics differ between direct measured samples and self report samples.

Comparing self-reported BMI from the Behavioural Risk Factor Surveillance System (BRFSS) with the directly measured data from the NHANES study revealed that the former underestimated the prevalence of obesity by 9.5% [39]. A recent study validating self-reported data from Spain showed that self-reported BMI led to moderate underestimation [40].

Several recent reviews have addressed the distribution and prevalences of obesity in Europe. These publications largely confirm our findings with respect to the regional distribution of obesity prevalences, including the east-west gradient and the lower prevalences observed in the Scandinavian countries. However, the overall prevalences reported in these publications were lower than those seen in our analysis and do not reflect recent developments in the Mediterranean countries [41]. The pan-EU study of obesity [31] conducted by the Institute of European Food Studies (IEFS) included over 15,000 individuals and also showed lower prevalences in data based on self-reported obesity. Reviews that include self-reported weight and height may thus underestimate obesity prevalence.

The limited validity in self-report estimates should be taken into consideration when using these data to make decisions concerning public health recommendations.

## Conclusion

In summary, although a variety of data on obesity and associated morbidity in Europe are available, there is considerable variation in the methods used by the different surveys. Improving the comparability of obesity-related prevalence data should remain a top priority in future research [42]. Moreover, developing effective and long-lasting therapeutic and preventive strategies will require acknowledging that obesity in Europe is a serious medical disorder.

## Competing interests

The authors declare that they have no competing interests.

## Authors' contributions

AB drafted the manuscript, TP has made substantial contributions to conception of the manuscript and interpretation of data, TR has made substantial contributions to acquisition of data and analysis, CMA and AMS have been involved in critically revising the manuscript and SNW participated in the study design and helped to draft the manuscript. All authors read and approved the final manuscript.

## Pre-publication history

The pre-publication history for this paper can be accessed here:


